# Pharmacological and Pathological Effects of Mulberry Leaf Extract on the Treatment of Type 1 Diabetes Mellitus Mice

**DOI:** 10.3390/cimb45070343

**Published:** 2023-06-29

**Authors:** Liru Luo, Wei Fan, Jingping Qin, Shiyin Guo, Hang Xiao, Zhonghai Tang

**Affiliations:** 1 College of Food Science and Technology, Hunan Agricultural University, Changsha 410128, China; 2 Hunan Engineering Technology Research Center for Rapeseed Oil Nutrition Health and Deep Development, Changsha 410128, China; 3 College of Bioscience and Biotechnology, Hunan Agricultural University, Changsha 410128, China

**Keywords:** mulberry leaf extract, type 1 diabetes mellitus, biochemical parameters, metformin, pharmacological effect, pathological effect

## Abstract

This study investigated the pharmacological and pathological effects of aqueous mulberry leaf extract on type 1 diabetes mellitus mice induced with an intraperitoneal injection of streptozotocin (STZ). Diabetic mice were randomized into six groups: control (normal group), model, metformin-treated mice, and high-dose, medium-dose, and low-dose mulberry. The mulberry-treated mice were divided into high-, medium-, and low-dose groups based on the various doses of aqueous mulberry leaf extract during gavage. The efficacy of the six-week intervention was evaluated by measuring levels of fasting plasma glucose, alkaline phosphatase, alanine aminotransferase, aspartate transaminase, blood urea nitrogen, gamma-glutamyl transferase, glucose, high-density lipoprotein cholesterol, lactate dehydrogenase, and low-density lipoprotein cholesterol and recording body weight. Results revealed that mulberry leaf extract exhibited an ideal hypoglycemic effect, and the high-dose group was the most affected. Histology analysis, glycogen staining and apoptosis detection were used to study the extract’s effects on the liver, kidney, and pancreatic cells of diabetic mice, enabling the assessment of its effectiveness and complications on a clinical and theoretical basis. It was shown that a certain concentration of aqueous mulberry leaf extract repaired the islet cells of type 1 diabetes mellitus mice, promoting normal insulin secretion. Herein, it was confirmed that mulberry leaf could be used to develop new hypoglycemic drugs or functional health food with broad applicability.

## 1. Introduction

Diabetes mellitus (DM) is familiar, associated with genetic and environmental factors, and is caused by insulin deficiency or decreased tissue and cell insulin sensitivity. It is a metabolic disorder characterized by chronically increased blood glucose levels, resulting in carbohydrate, fat, protein, water, and electrolyte metabolism disturbances. According to the International Diabetes Union, 463 million adults worldwide had diabetes in 2019, accounting for one-eleventh of adults worldwide, and the prevalence and incidence of diabetes are increasing worldwide [1,2]. Diabetes is the third most morbid disease after cardiovascular disease and cancer and is one of the three major public health challenges worldwide. Type 1 diabetes mellitus (T1DM)—also known as insulin-dependent diabetes mellitus—accounts for approximately 10–15% of patients with diabetes [3]. In patients with T1DM, the insulin-producing cells of the pancreas have been largely destroyed, greatly reducing the ability to produce insulin. An absolute lack of insulin will continuously raise the blood sugar level, resulting in diabetes [4]. Diabetes can damage multiple body systems. Hyperglycemia is the major clinical sign, and common symptoms include polydipsia, polyuria, polyphagia, and sarcopenia. With disease progression, chronic complications in multiple systems involving the liver, kidney, heart, optic reticular membrane, and nerves can occur, eventually leading to atherosclerosis, coronary atherosclerotic heart disease, hypertension, diabetic liver disease, diabetic nephropathy, diabetic retinitis, and diabetic peripheral neuropathy. Despite the availability of insulin preparations and several synthetic oral antidiabetic drugs, their toxicities and side effects are substantial. Therefore, finding and developing natural products with hypoglycemic effects are essential [5,6].

Mulberry (*Morus alba* L.), also known as Jing mulberry, mulberry tree, and yellow mulberry, is mainly found in China, Japan, and Korea. It is one of the medicinal and edible (dual-purpose) plants announced by the Ministry of Health, China. It is highly medicinal and nutritive; thus, it is frequently used as a therapeutic agent to treat fever, protect the liver, and control blood pressure [7]. Mulberry leaves contain polysaccharides, flavonoids, alkaloids, volatile oils, and other active components with hypoglycemic, hypolipidemic, antioxidant, anti-aging, and other effects [8,9,10,11]. In China, mulberry leaf has been clinically used as a traditional Chinese medicine for thirst (equivalent to diabetes in modern medicine) since ancient times [12]. Many scholars at home and abroad have reported on diabetes treatment with mulberry leaves, mostly focusing on the hypoglycemic effect of polysaccharides and flavonoids in mulberry leaves [13,14,15,16]. Iminosugars and sugar derivatives are effective antidiabetic agents [17,18,19]. Modern pharmacological studies have revealed that mulberry leaves are rich in the alkaloid 1-deoxynojirimycin (DNJ), which is a prototypical iminosugar compound [20]. DNJ can effectively inhibit the activity of enzymes that decompose carbohydrates, prevent glucose absorption, and reduce blood sugar levels [21].

In this study, a mouse model of T1DM induced by the intraperitoneal injection of streptozotocin was used to observe the hypoglycemic effect of mulberry leaf water extract. In the experiment, levels of fasting plasma glucose, alkaline phosphatase (ALP), alanine aminotransferase (ALT), aspartate transaminase (AST), blood urea nitrogen (BUN), gamma-glutamyl transferase (GGT), glucose (GLU), high-density lipoprotein cholesterol (HDL-C), lactate dehydrogenase (LDH), and low-density lipoprotein cholesterol (LDL-C) were measured, and body weight was recorded. Further, the effect of mulberry leaf water extract on blood glucose levels and lipid metabolism in diabetic mice was investigated. After the experiment, the pathological section analysis, glycogen staining (periodic acid Schiff [PAS]) and apoptosis detection method (TUNEL) were used to study the effect of the mulberry leaf extract on the liver, kidney, and pancreatic cells of diabetic mice. It was known that the high-dose mulberry extract could repair the liver and pancreas of STZ-induced T1DM mice, but the kidney restoration was not obvious. This experiment establishes a foundation for using mulberry leaf’s secondary metabolites in the comprehensive prevention and treatment of diabetes mellitus and its complications, both in theory and in clinical applications.

## 2. Materials and Methods

### 2.1. Preparation of the Experimental Materials

**Figure 1:**
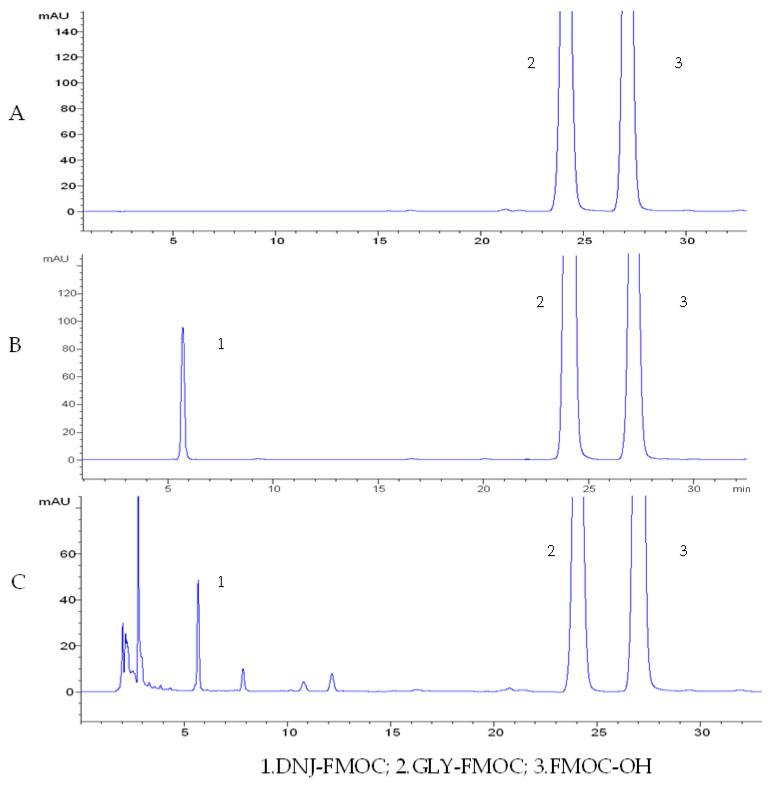
Blank (**A**), DNJ chromatogram of the standard sample (**B**) and mulberry leaf extract (**C**) with HPLC.

The aqueous mulberry leaf extract (1:10): Mulberry leaves were collected from the National Silkworm Research Center Changsha sub-center (China). After washing with cold water, the samples were dried at 50 °C, ground into 30-mesh powder using a Chinese medicine pulverizer, and stored at 0–4 °C until further use. After the powder was accurately weighed, pure water was added in a 1:25 ratio. Then, the sample was refluxed at 70 °C twice, for 90 min each time, and the extract was collected. The extract was concentrated using a rotary evaporator and then freeze-dried into powder. The content of the main active ingredient in the aqueous mulberry leaf extract of polysaccharide was 1.69% (Anthranone-sulfuric acid method), and the content of 1-Deoxynojirimycin (DNJ) was 0.26% (PMP-HPLC) (Figure 1).

Specific pathogen-free Kunming-based mice, with body weights of 22–26 g, were purchased from Hunan Slack King experimental animal Co., Ltd., China. (certificate number: SCXK (Hunan) 2009-0004).

All animal experiments were carried out under the scheme approved by the animal experiment welfare and the Ethics Inspection Committee of Hunan Agricultural University. Efforts were made to minimize experimental animal pain.

### 2.2. Reagents and Instruments

STZ (Sigma Company, Steinheim am Albuch, Germany), metformin (Gehuazhi Pharmaceutical Co., Ltd., Shanghai, China), paraffin (Shanghai Bioengineering Co., Ltd., Shanghai, China), PAS stain reagent (Shanghai yuanye Bio-Technology Co., Ltd., Shanghai, China), and paraformaldehyde (Beijing Chemical Reagent Factory, Beijing, China) were purchased. A blood glucose test strip and glucometer (U.S. Grace Medical company, Columbia, MD, USA), superoxide dismutase (SOD) kit (Nanjing Bioengineering Institute, Nanjing, China), mouse insulin (Ins) ELISA test kit (U.S. R & D company, America), TUNEL test kit (Roche), light microscope (OLYMPUS TOKYO JAPAN, Tokyo, Japan), Olympus Optical microscope photography system (OLYMPUS Company, Tokyo, Japan), microtomes (Leica Company, Munich, Germany), cryogenic refrigerator (Hitachi Company, Tokyo, Japan), Superclean bench (Suzhou Hongrui Company, Jiangsu, China), Agilent 8453 type UV-visible spectrophotometer (U.S. Agilent, Santa Clara, CA, USA), FD-1B- 50 vacuum freeze dryer (Beijing Boyi Kang Experimental Equipment Co., Ltd., Beijing, China), MB-530 Enzymatic Standard instrument (Shenzhen Huisong Technology Development Co., Ltd., Shenzhen, China), and Beckman Chemical Analyzer (U.S. BECKMAN Company, Model CX4, Brea, CA, USA) were used.

### 2.3. The Diabetic Mouse Model

The Hunan Agricultural University Laboratory Animal Ethics Committee provided ethical approval for using and handling mice. The mice were raised in the standardized animal room (22 °C ± 2 °C) and adapted to the environment for 1 week. The mice had ad libitum access to food and water, which were changed day and night. The animal room was well-ventilated, and the relative humidity was 40–70%. Natural light was allowed into the room, avoiding direct sunlight. The mice drank distilled water and were cushioned using dry, clean, fine shavings. We changed the water and cushions once daily.

STZ was prepared with 0.1 mol/L citric acid buffer (pH 4.3) to form 10 mg/mL [22].

After the male Kunming mice were fasted for 16 h, they were administered a single intraperitoneal injection of STZ (160 mg/kg), and fasting blood glucose was measured in the tail blood after 3 and 10 days. The mice with fasting blood glucose of ≥16.7 mmol/L were considered T1DM model mice [23].

### 2.4. Grouping, Doses, and Administration

Diabetic mice were randomized into control (healthy mice), model, and metformin-treated (Metformin) groups. The mulberry group was divided into high-, medium-, and low-dose groups based on the various doses of the aqueous mulberry leaf extract during gavage, with 20 mice in each group. The body weights and fasting blood glucose levels were measured before the experiment. Gastric treatment in each group was as follows:

Metformin group: metformin hydrochloride was intragastrically administered to diabetic rats at 270 mg/kg daily.

High-dose group: diabetic mice received 1200 mg/kg/day of the aqueous mulberry leaf extract;

Medium-dose group: diabetic mice received 600 mg/kg/day of the aqueous mulberry leaf extract;

Low-dose group: diabetic mice received 300 mg/kg/day of the aqueous mulberry leaf extract.

For 42 consecutive days, 1 g was administered once daily. The control (normal group) and model groups were administered the same volume (10 mL/kg) of distilled water. Except for the testing requirements, all experimental mice drank distilled water and fed ad libitum. The changes in blood sugar level and body weight of mice in each group were recorded during the intragastric administration.

### 2.5. Measurement of Plasma Glucose

Blood samples for measuring fasting blood glucose were collected from the experimental mice at day 0, 10, 20, 30, and 40. After fasting for 10–12 h, blood was withdrawn from the mice’s tail vein, and fasting blood glucose was immediately measured using a blood glucose meter and test strip.

### 2.6. Measurement of Antioxidant and Biochemical Indexes

Whole blood was collected from the mouse’s orbit after the last intragastric administration, and serum was segregated and stored in a refrigerator at −20 °C for detection.

Serum insulin, SOD, and malonic dialdehyde (MDA) levels were determined using ELISA.

The levels of ALP, ALT, AST, BUN, GLU, cholesterol (CHO), triglyceride (TG), total serum cholesterol (TC), LDH, HDL-C, and LDL-C in serum were measured with a Beckman CX4 biochemical analyzer.

### 2.7. The Effect of Mulberry Leaf Extract on Glucose Tolerance in Diabetic Mice

After forming the groups for 2 days, the animals were fasted for 12 h. After the third day of administration for 30 min, glucose was administered intragastrically (2 g/kg); the blood glucose levels at 0, 0.5, 1, and 2 h were measured using blood from the rat tail vein; and the area under the curve (AUC) of the blood glucose was calculated as follows [24],

AUC (mmol·h/L) = 0.25 A + 0.5 B + 0.75 C + 0.5 D(1)

(A, B, C, and D correspond to 0, 0.5, 1, and 2 h blood glucose concentrations, respectively.)

### 2.8. Pathological Examination Methods

The glass slides and utensils were soaked in acid and alkali in advance, washed thoroughly, and dried at 160 °C for 2 h. Diabetic mice were killed after 6 weeks of treatment. The pancreatic tissues were extracted immediately, placed in 4% formaldehyde for 24 h, rinsed thoroughly with distilled water, and gradually placed in 70–100% ethanol dehydration. Then, dimethylbenzene was used to make the tissues transparent, and tissues were soaked in paraffin. The tissues were embedded and sliced (5-μm thick), and the slices were pasted on a 0.01% polylysine-coated slide and baked in an oven at 60 °C for 2 h.

The pathological changes in the fixed pancreas, kidney, and liver tissues were observed under a light microscope. Red shades were observed using an optical microscope after the PAS glycogen staining. The kidney and liver tissues were analyzed using the TUNEL pathological section. Apoptosis was observed, and its rate was calculated using Image Pro Plus 6 software.

### 2.9. Statistical Analysis

Data are expressed as mean ± standard deviation (x ± s), and the significant differences between two groups were compared using SPSS18.0 statistical analysis software. Data were considered significant when *p* < 0.05 and extremely significant when *p* < 0.01.

## 3. Results

### 3.1. Effect of the Aqueous Mulberry Leaf Extract on Blood Glucose in T1DM Mice

**Figure 2:**
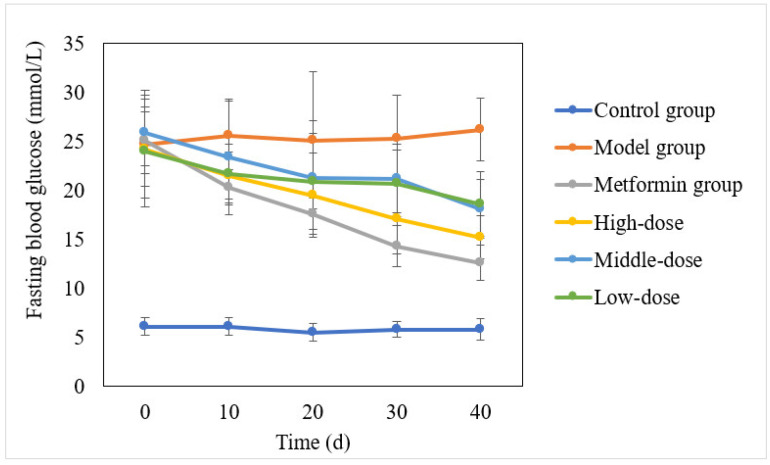
Hypoglycemic effect of the aqueous mulberry leaf extract on T1DM mice.

The blood glucose levels of each group were monitored to investigate the hypoglycemic effect of the aqueous mulberry leaf extract on diabetic mice. After 10 days, the aqueous mulberry leaf extract was effective, with an effect slightly weaker than that of metformin hydrochloride (Figure 2).

### 3.2. The Effect of the Aqueous Mulberry Leaf Extract on Serum Lipid (HDL-C, LDL-C, TG) and Insulin Levels in T1DM Mice

**Figure 3:**
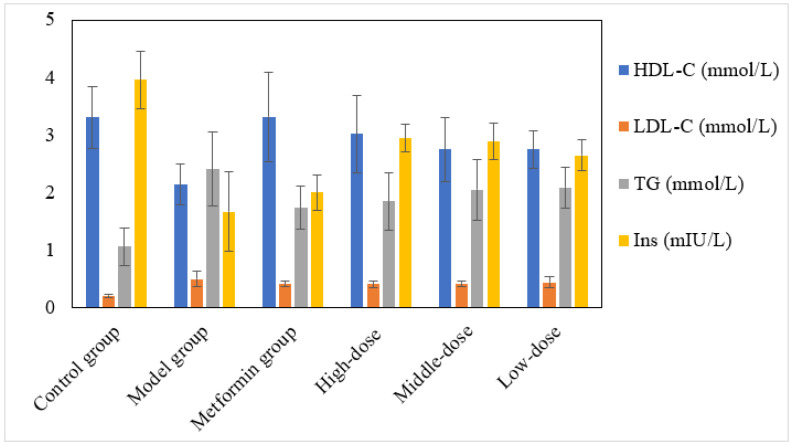
Effect of the aqueous mulberry leaf extract on serum lipids (HDL-C, LDL-C, and TG) and serum insulin levels in T1DM mice.

The aqueous mulberry leaf extract and metformin hydrochloride were dose-dependently effective for lipid metabolism disorder (Figure 3). However, the aqueous mulberry leaf extract was superior to the metformin regarding serum insulin.

### 3.3. The Effect of the Aqueous Mulberry Leaf Extract on Glucose Tolerance in T1DM Mice

Table 1 presents the changes in blood glucose concentrations and areas under the glucose curve in each group during the glucose tolerance test. The highest blood glucose value was observed at 0.5 h after the intragastric glucose administration. Metformin hydrochloride’s onset was 0.5 h, while the mulberry leaf extract’s onset was 1 h. Compared with mice in the model group, those in the mulberry-treated groups exhibited decreased blood glucose concentrations at 1 h and 2 h; blood glucose concentration significantly decreased in the high-dose group (*p* < 0.01). In contrast, glucose metabolism improved after treatment with the high/medium doses of mulberry extract. Thus, the extract could reduce blood glucose and improve glucose tolerance in diabetic mice [25,26].

### 3.4. The Effects of the Aqueous Mulberry Leaf Extract on Alkaline Phosphatase in T1DM Mice

**Figure 4:**
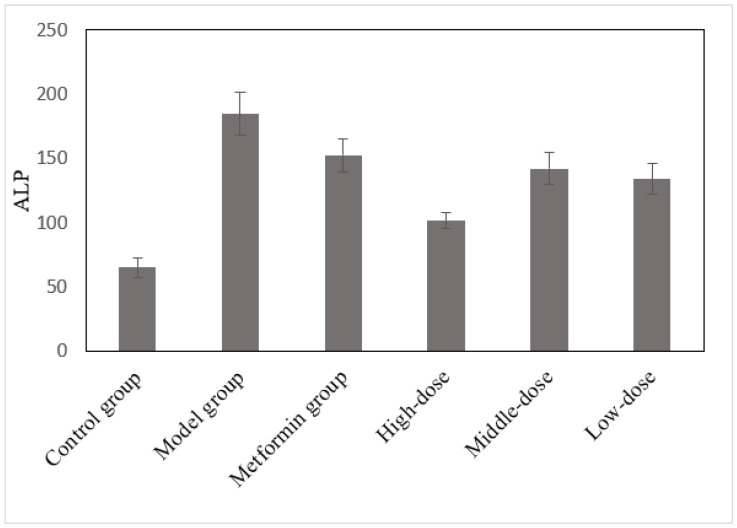
The effects of the aqueous mulberry leaf extract on ALP in T1DM mice.

ALP levels in each group were assessed using a Beckman CX4 biochemical analyzer (Figure 4). Using the least significant difference (LSD) method, with the ALP level as the dependent variable, other groups were compared with the model group (Table S1).

Figure 4 and Table S1 indicate that the ALP of the mulberry and metformin groups significantly decreased. In contrast, that of the model group remained at a high level. The high-dose group had an extremely significant change in ALP (*p* < 0.001), and the low-dose group reached a significant difference level (*p* < 0.01). The difference in the medium-dose group was significant, whereas ALP decreased in the metformin group; however, it was not significant (*p* > 0.05).

ALP is an enzyme with low substrate specificity that can hydrolyze various phosphate monoester compounds in an alkaline environment. It is associated with substance absorption and transportation. Clinically, renal or liver function impairment leads to elevated ALP levels, and the mulberry leaf extract has some curative effects on renal and liver insufficiency in T1DM mice [27,28].

### 3.5. The Effects of the Aqueous Mulberry Leaf Extract on ALT in T1DM Mice

**Figure 5:**
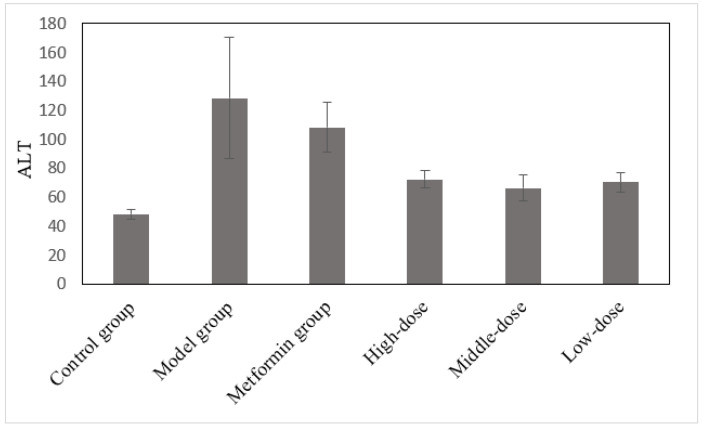
The effects of the aqueous mulberry leaf extract on ALT in T1DM mice.

After 4 weeks of treatment, serum ALT level was measured using a Beckman CX4 biochemical analyzer (Figure 5). These experimental data were analyzed through ANOVA, using the least significant difference (LSD). The ALP level was the dependent variable in comparing the treatment and model groups (Table S2).

ALT levels in the control group were significantly lower than those in the model group (*p* < 0.01), and the ALT levels in the mulberry and metformin groups decreased (Figure 5 and Table S2). The difference between the treatment and model groups was significant (*p* < 0.05). The metformin group had decreased ALT; however, it was not significant (*p* > 0.05). Figure 5 and Table S2 reveal that the liver of T1DM mice was damaged, leading to increasing ALT levels. The liver cell injury caused ALT to enter the bloodstream, increasing serum ALT levels. After treatment with mulberry leaf extract for 4 weeks, the injured hepatocytes were repaired to a certain extent. However, ALT could not be restored to the control group’s level; thus, the extract’s effect on hepatocyte repair in diabetic mice was not obvious.

### 3.6. The Effects of the Aqueous Mulberry Leaf Extract on AST in T1DM Mice

**Figure 6:**
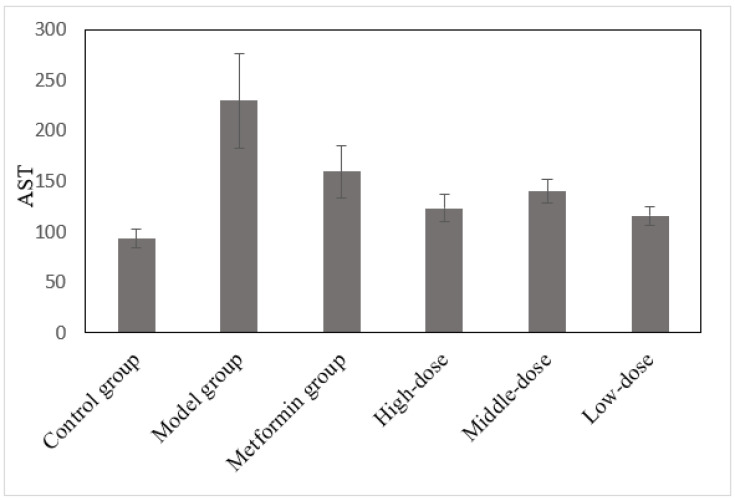
The effects of the aqueous mulberry leaf extract on AST in T1DM mice.

After 6 weeks of treatment, the serum aspartate transaminase (AST) was measured using a Beckman CX4 biochemical analyzer (Figure 6). These experimental data were analyzed through ANOVA, using the LSD. AST level was a dependent variable in comparing the treatment and model groups (Table S3).

AST, like ALT, can reflect the liver’s health status [29]. When the liver is damaged by inflammation, necrosis, or poisoning, AST is released from the liver cells and enters the bloodstream [30]. Figure 6 and Figure S3 reveal that the AST level in the model group increased significantly (*p* < 0.001) compared to the control group. In contrast, the AST level in the mulberry-treated groups decreased significantly (*p* < 0.01). The metformin group had decreased AST, which was significant (*p* < 0.05). Thus, mulberry leaf extract and metformin can repair liver damage to some extent. However, metformin’s repair effect was weaker than that of mulberry leaf extract.

### 3.7. The Effects of the Aqueous Mulberry Leaf Extract on BUN in T1DM Mice

**Figure 7:**
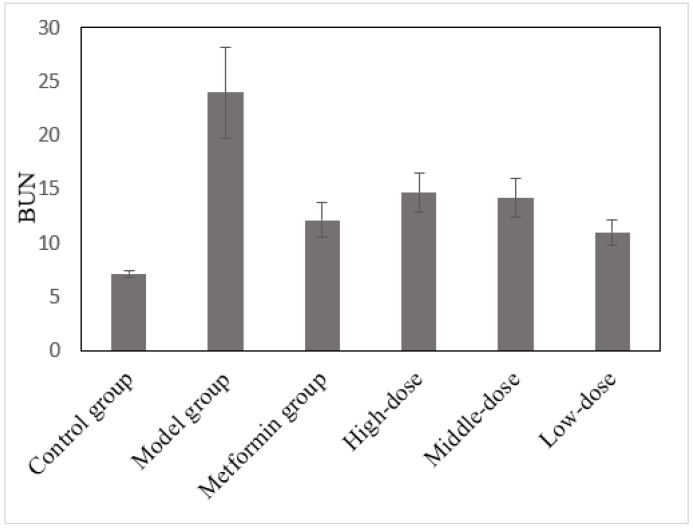
The effects of the aqueous mulberry leaf extract on BUN in T1DM mice.

Urea is an end product of protein metabolism that is mainly filtered by the glomerulus and excreted into the urine. In the early renal damage stage, BUN remained within the normal range. However, when the glomerulus filterability decreases to < 50%, the BUN concentration increases rapidly, which is associated with glomeruli injury and primary kidney function [31,32]. BUN levels in each group were investigated using a Beckman CX4 biochemical analyzer (Figure 7). The experimental data in Figure 7 were analyzed using ANOVA and LSD. The BUN level was the dependent variable in comparing the treatment and model groups (Table S4).

The BUN levels of diabetic mice in the model group increased significantly (*p* < 0.001), and those in the treatment and metformin groups decreased (Figure 7 and Figure S4). The BUN level changes in the metformin and low-dose mulberry groups were very significant (*p* < 0.001), whereas those in the high- and medium-dose mulberry groups were significant (*p* < 0.01). Therefore, the relationship between the treatment and glomerular repair is uncertain. However, combined with the blood glucose levels in each group, mulberry leaf extract and metformin can increase glycogen stores in T1DM mice and improve the BUN level.

### 3.8. The Effects of the Aqueous Mulberry Leaf Extract on CHO in T1DM Mice

**Figure 8:**
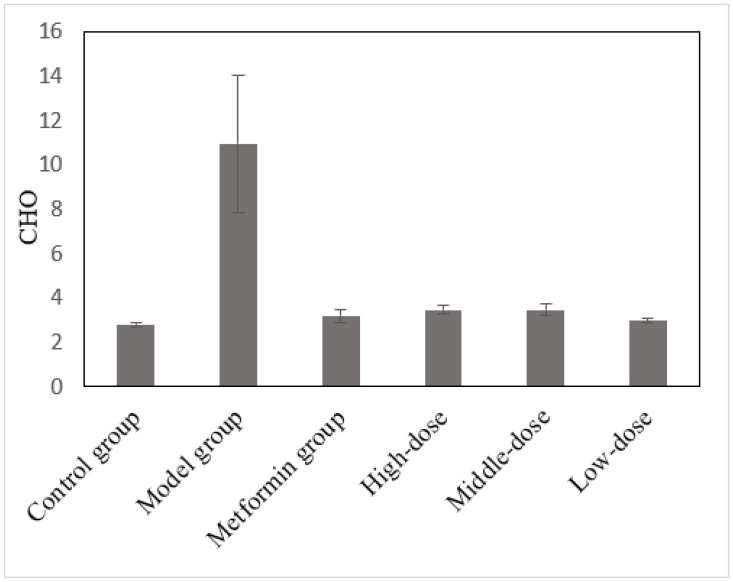
The effects of the aqueous mulberry leaf extract on CHO in T1DM mice.

The total CHO levels in each group were measured at the end of the experiment (Figure 8). These data were analyzed using the LSD method, with CHO as the dependent variable, and compared with the model group (Table S5).

Statistics reveal that the CHO level of diabetic mice in the model group was much higher than that in the control group (*p* < 0.001). After the treatment, the CHO levels in the metformin and mulberry groups decreased significantly (*p* < 0.001) and were maintained within the standard. Atherosclerosis, nephrotic syndrome, hepatocellular jaundice, obstructive jaundice, and diabetes can cause an elevated CHO [33]. In this experiment, the CHO in the mulberry and metformin groups was maintained at a normal level. This was because the metformin and mulberry leaf extract decreased blood glucose levels and reduced catabolism.

### 3.9. The Effects of the Aqueous Mulberry Leaf Extract on Lactate Dehydrogenase in T1DM Mice

**Figure 9:**
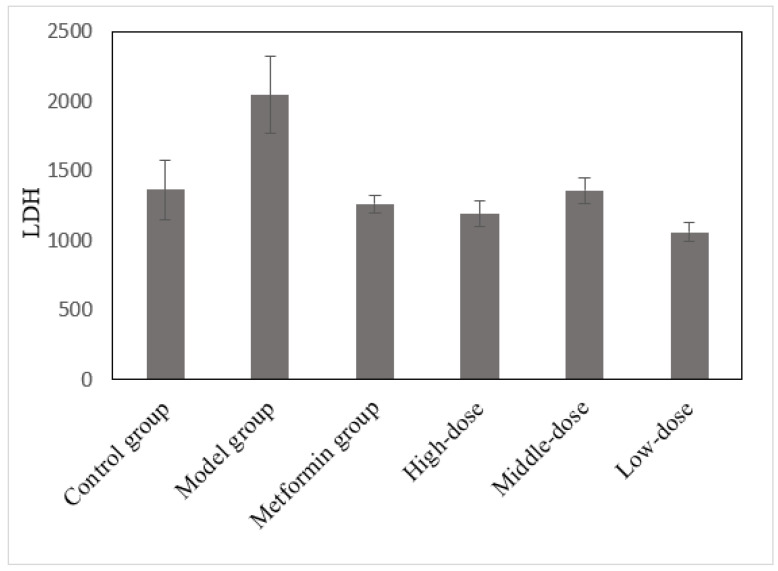
The effects of the aqueous mulberry leaf extract on LDH in T1DM mice.

LDH is an important glycolytic enzyme, and liver cell injury can cause its release and entry into the serum [34]. However, when LDH-rich tissue or cells, such as the myocardial, renal, intestinal, or blood cells, are damaged, it also can lead to increased LDH levels [35,36]. The LDH levels in each group were measured using a Beckman CX4 biochemical analyzer (Figure 9). These experimental data were analyzed through ANOVA using LSD. The LDH level was considered the dependent variable in comparing the mulberry and model groups (Table S6).

Figure 9 and Figure S6 reveal that the LDH in the model group remained high at the end of the experiment. The LDH levels in the mulberry and metformin groups decreased after treatment. Furthermore, the changes in the LDH levels in the metformin, high- and low-dose groups were very significant (*p* < 0.001). The changes in the medium-dose group were significant (*p* < 0.01). Based on the above data, sustained hyperglycemia in diabetic mice damaged the liver, kidney, and other metabolic organs, increasing the LDH level. Nevertheless, after treatment with the effective drug, the blood glucose level was controlled, and the LDH level was reduced in diabetic mice.

### 3.10. The Effects of the Aqueous Mulberry Leaf Extract on β-2-Microglobulin in T1DM Mice

**Figure 10:**
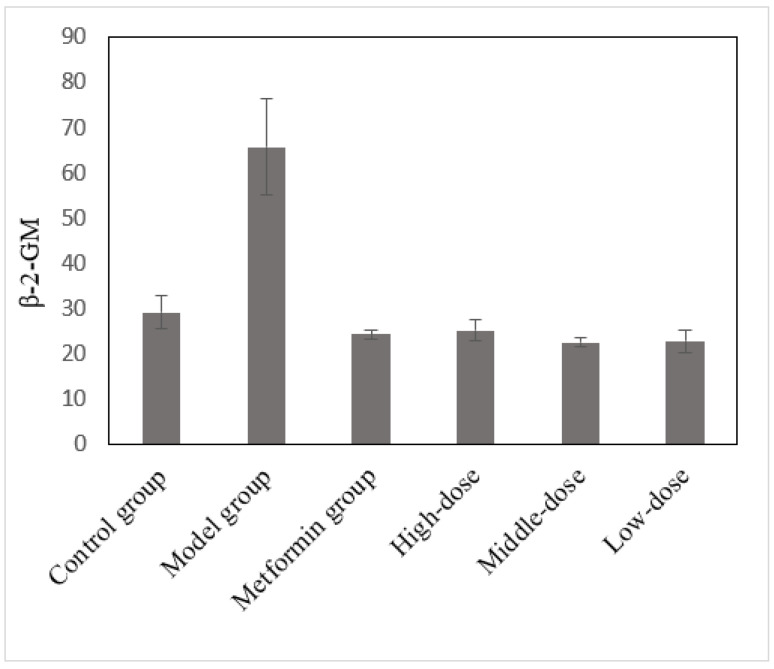
The effects of the aqueous mulberry leaf extract on β-2-GM in T1DM mice.

β-2-MG is a low molecular weight protein. Proximal tubule damage and increased β-2-MG synthesis can increase serum β-2-MG levels [37,38]. After 6 weeks of treatment, the serum β-2-MG levels in each group were measured using a Beckman CX4 biochemical analyzer (Figure 10). Using the LSD method and β-2-MG level as the dependent variable, the model and mulberry groups were compared (Table S7).

Serum β-2-MG levels in the model group increased significantly (*p* < 0.001). In addition, high blood glucose levels in diabetic mice increased β-2-MG synthesis. After the treatment, the blood glucose levels decreased, reducing β-2-MG synthesis (*p* < 0.001).

### 3.11. Effect of the Aqueous Mulberry Leaf Extract on Superoxide Dismutase Activity and Malonic Dialdehyde Level in Serum of T1DM Mice

The mulberry leaf extract significantly increased the SOD activity (more with the high-dose mulberry than with metformin) (Table 2). The MDA levels in the treatment and metformin groups were remarkably reduced (*p* < 0.01). The results showed that the aqueous mulberry leaf extract increased the reactive oxygen species scavenging, reduced free radical injury, and inhibited lipid peroxidation.

### 3.12. Effects of the Aqueous Mulberry Leaf Extract on the Liver of Diabetic Mice

**Figure 11:**
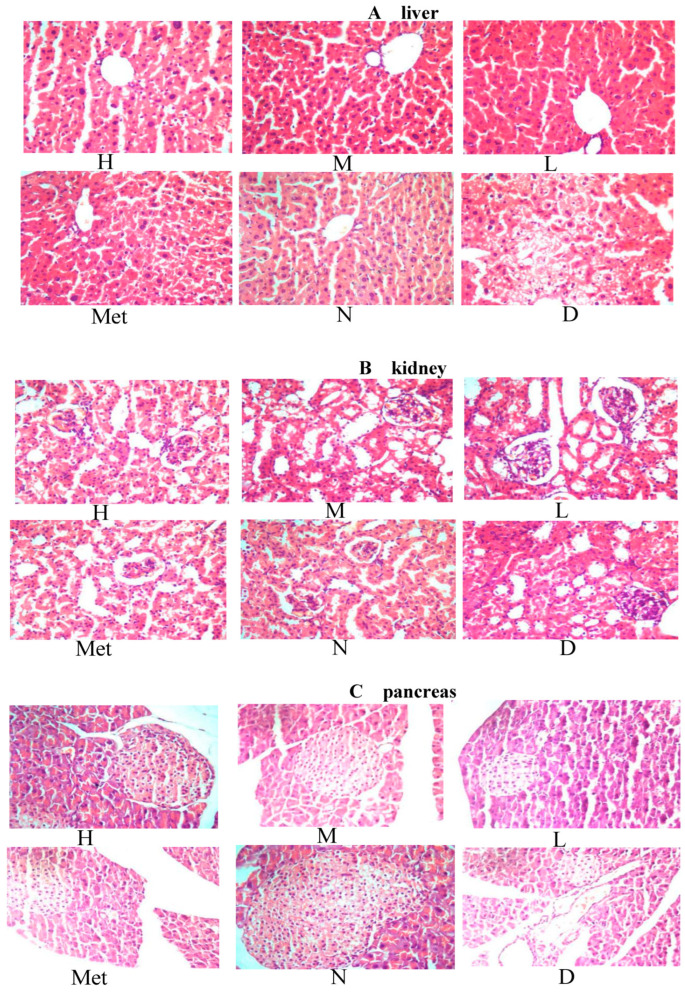
Pathological sections in diabetic mice ((**A**): Liver, (**B**): Kidney, (**C**): Pancreas). (High-dose group (H), Medium-dose group (M), Low-dose group (L), Metformin group (Met), Normal group (N), Model group (D)).

The pathological results for the liver in diabetic mice are presented in Figure 11A. The hepatocytes in the model group were disordered, indicating focal lytic necrosis. Moreover, many vacuoles of various sizes appeared in the cytoplasm, and the nucleus was squeezed aside. The arrangement of hepatocyte cords was more disordered in the metformin group than in the normal group. However, it was more regular than that in the model group. The groups treated with medium- and low-dose mulberry leaf water extract had a more regular hepatic cord arrangement than the metformin group. However, the central venous dilation remained obvious. The high-dose group had a worse hepatic cord arrangement than the medium-dose group. The central venous expansion was not obvious, but the slight focal lytic necrosis was visible.

**Figure 12:**
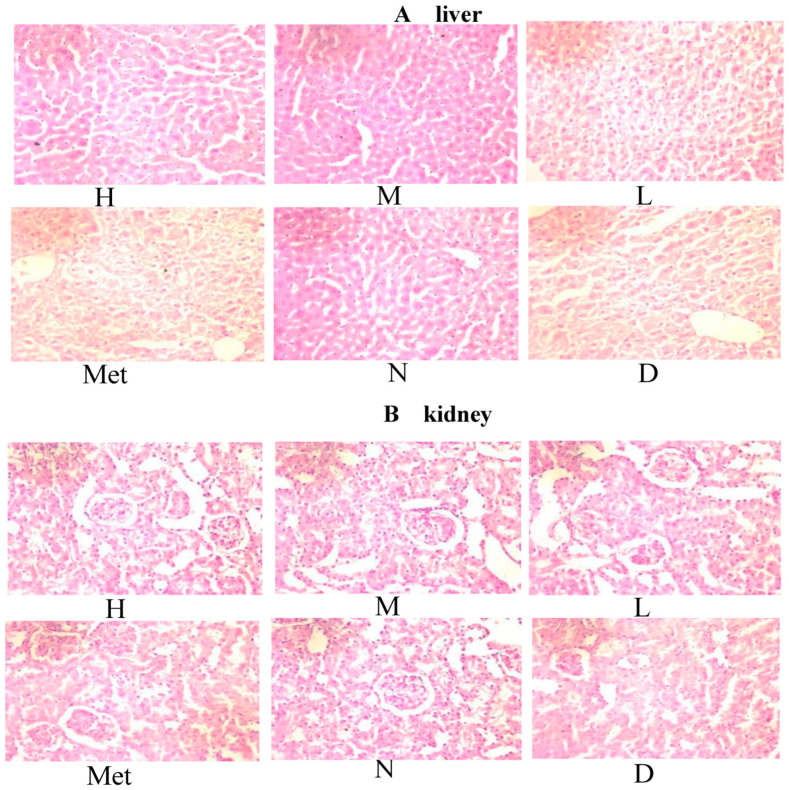
PAS staining pictures in diabetic mice ((**A**): Liver, (**B**): Kidneys) (High-dose group (H), Medium-dose group (M), Low-dose group (L), Metformin group (Met), Normal group (N), Model group (D)).

Figure 12A illustrates the results of PAS staining of the liver in each group. The model group had less liver glycogen, and lipid accumulation in the liver was caused by increased blood glucose, which destroyed the hepatocytes’ structure and affected their function, affecting glycogen accumulation in the liver. Moreover, glycogen level was not significantly increased in the metformin group. However, the organizational structure was more regular in this group than in the model group. In the mulberry groups, the medium-dose group had the most liver glycogen distribution, and the color and depth distribution matched that of the normal group. The high-dose group was the second, and the low-dose group was the most light-colored. However, the glycogen distribution in the liver cells was more extensive and deeper in the three mulberry groups than in the metformin group. This was consistent with the results of routine pathological sections.

**Figure 13:**
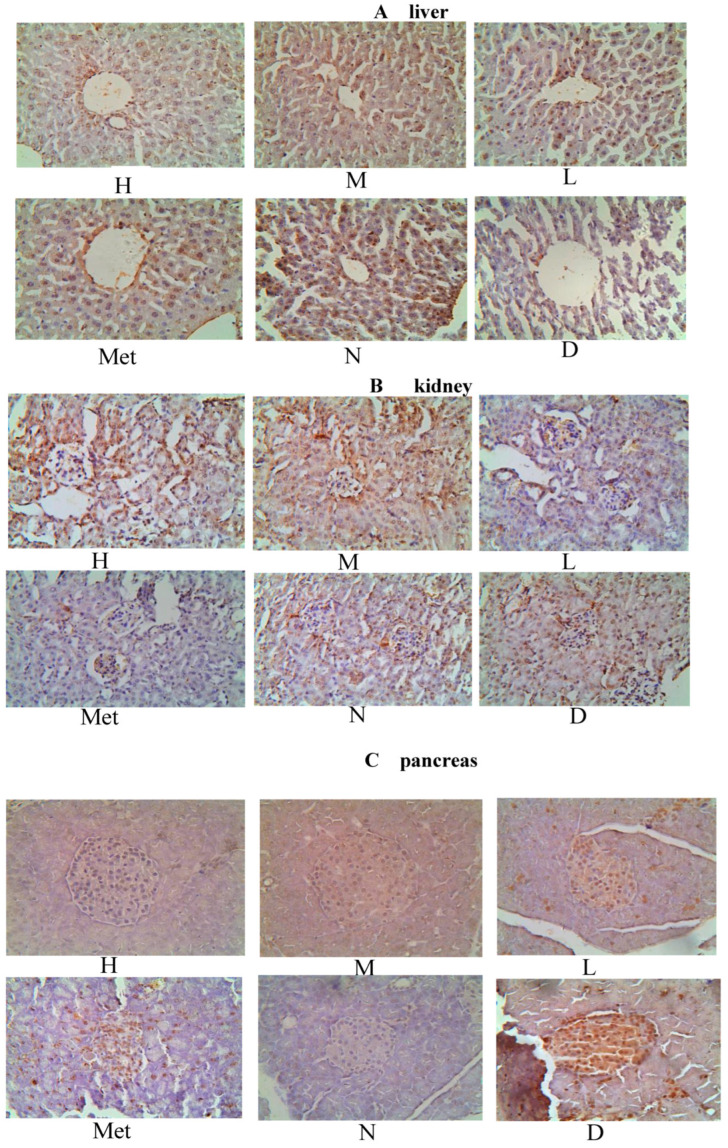
Apoptosis (TUNEL) test in diabetic mice ((**A**): Liver, (**B**): Kidney, (**C**): Pancreas) (High-dose group (H), Medium-dose group (M), Low-dose group (L), Metformin group (Met), Normal group (N), Model group (D)).

The TUNEL test results (Figure 13A) were analyzed using Image pro plus 6.0 software. The apoptosis rate of hepatocytes in the model group reached 71.43%. The cell distribution in the metformin group was regular; however, the cell apoptosis rate was up to 54.55%, comparable to that in the low-dose mulberry group (56.25%). The liver cell distribution in the medium-dose mulberry group (apoptosis rate: 30.77%) was similar to but not as regular as that in the normal group. The cell apoptosis rate in the normal group was 21.05%, within the hepatocyte apoptosis rate range in normal mice. The cell apoptosis rate in the high-dose group was 36.36%, slightly higher than that in the medium-dose group.

Comprehensive common pathological sections, PAS staining, and TUNEL cell apoptosis assay results were obtained. Metformin inhibited the hepatocyte damage caused by hyperglycemia but could not repair the damaged liver cells. In the aqueous mulberry leaf extract group, treating STZ-induced diabetes in mice using the medium dose was the most appropriate for liver cell recovery, followed by the high dose. In contrast, the low dose had no obvious effect. This implies that the appropriate concentration of the aqueous mulberry leaf extract can repair the hepatocytes in STZ-induced I-diabetic mice and strengthen the liver’s ability to store glycogen.

### 3.13. Effect of Aqueous Mulberry Leaf Extract on Kidney in Diabetic Mice

The pathological findings for the kidney in diabetic mice are illustrated in Figure 11B. In the model group mice, renal tissue slices showed a significant increase in the volume of kidney glomeruli relative to the normal group. The main reason is that the glomerular filtration globulin and the protein in the blood are accumulated in the renal tissue of mice with glycosuria, increasing the kidney glomeruli. In the normal group, the thickness of the glomerular basement membrane was even, the endothelial and Sertoli cells were intact, and the podocytic processes were neatly arranged. Significant differences were observed in the basement membrane thickness of different glomerulus parts among the three groups. The low-dose group had less glomerular basement membrane thickening than the model group. The medium-dose group had diffuse thickening of the basement membrane. The swelling of the cells in the metformin and the high-dose groups improved, with their glomerular volume being closest to that of the normal group. However, the hepatocyte distributions in the metformin and normal groups were more uniform, while those in the high-dose group were slightly worse.

The results of renal PAS staining are illustrated in Figure 12B. In the model group, the renal arteriole wall of the mice was significantly thickened, with lumen expansion and epithelial cell degeneration. More inflammatory cell infiltration was observed in the intercellular substance, the interstitial area widened, renal tubules had obvious vacuolar degeneration, and proximal convoluted tubules of glomeruli had dark red plaques or glycogen deposition—all typical manifestations of renal lesions in diabetic mice. The infiltration of interstitial inflammatory cells into renal cells was lower in the metformin group than in the model group. Thus, renal epithelial cell disease was relieved, and renal epithelial cell lesions were clearly reduced. The high-, medium-, and low-dose aqueous mulberry leaf extract and metformin groups had PAS-positive glycogen deposition, close to that of the normal group.

The apoptosis findings from renal biopsy (TUNEL) are illustrated in Figure 13B. We used Pro Plus 6 statistical software for statistical analysis and observed that the high-, medium-, and low-dose groups had cell apoptosis rates of 36.36%, 31.25%, and 45.46%, respectively. Based on the apoptosis rate, the inhibitory effect of aqueous mulberry leaf extract on apoptosis was less than that of metformin (27.58%). In the model group, the apoptosis rate in mice was 45.45%, implying substantial renal cell denaturation and necrosis. The results were consistent with those of conventional slices and PAS staining analysis. Cell apoptosis rates were higher in the metformin and aqueous mulberry leaf extract groups than in the normal group (12.90%). In addition, the apoptosis rate was significantly lower in the low-dose group than in the model group. The main reason is the drug and medium. The high-dose aqueous mulberry leaf extract inhibited cell apoptosis. In contrast, the low dose had no therapeutic effect on diabetic mice, and whether or not it had to reach a specific concentration remained to be verified.

### 3.14. Effect of the Mulberry Leaf Extract on the Kidney of Diabetic Mice

The pathological findings for kidneys in diabetic mice are presented in Figure 11C. The model group maintained hyperglycemia for a long time. It affected the growth and differentiation of islet β cells, and the ability to synthesize insulin was reduced—from early compensatory hyperplasia and hypertrophy of the islet cells to atrophy and diminution. In the low-, medium-, and high-dose mulberry groups, some islet cell repair activity was observed, which was gradually enhanced. In the metformin group, the islet cells had no atrophy and decreased size because of the blood sugar level control in diabetic mice. Therefore, no fundamental repair of the compensatory hyperplasia and hypertrophy of islet cells was observed in the modeling process.

The results for pancreas cell apoptosis are presented in Figure 13C. The model group had loosely organized islet cells. Generally, long-term hyperglycemia is believed to accelerate β cell apoptosis. While the apoptosis rate exceeds the regeneration rate, pancreatic islets will atrophy. The image application Image Pro Plus 6.0 software was used for statistical analysis. The apoptosis rate of the model group was high (89.65%), exceeding that of the normal group (8.47%). The metformin group’s pancreatic cell apoptosis rate decreased to 67.95%, similar to that of the low-dose mulberry group (67.21%), and the high-dose group exhibited a good level of apoptosis inhibition (20.43%), followed by the medium-dose group (40.91%).

## 4. Discussion

In this experiment, male mice were selected for single intraperitoneal STZ injection. The islet β cells were damaged, the insulin secretion of islet β cells was reduced, and the I-diabetic animal model was successfully established. The aqueous extract of mulberry leaves has an ideal hypoglycemic effect on T1DM model mice (Table S8). Its effect is moderate and slow compared with that of metformin hydrochloride. At the end of the experiment, aqueous mulberry leaf extract remained effective, especially in the high-dose group.

In addition, mulberry leaf extract effectively improved the disorder in lipid metabolism in T1DM mice, enhanced reactive oxygen species scavenging ability, and reduced the level of MDA with cytotoxicity in vivo. Glucose tolerance assessments revealed that the aqueous mulberry leaf extract became effective when the blood glucose level of the T1DM mice had reached its peak; otherwise, it was moderately effective. Table 1 indicates that the aqueous mulberry leaf extract decreased the glucose AUC and increased the glucose tolerance of T1DM mice. Further, a dose–effect relationship was observed.

The serum insulin test results revealed that the etiology of T1DM is islet β cell destruction, which leads to insufficient insulin secretion and hyperglycemia. However, compared with the control group, the insulin levels in the mulberry-treated groups increased (*p* < 0.01).

Therefore, the aqueous mulberry leaf extract has a remarkable hypoglycemic effect on T1DM mice by improving glucose and lipid metabolism, enhancing scavenging of reactive oxygen species, and reducing the oxidative damage of free radicals on islet β cells. The mulberry extract might also promote the repair and proliferation of islet β cells.

The high-dose mulberry extract could repair the liver and pancreas of STZ-induced T1DM mice. Further, the kidney restoration was not obvious. The medium dose had a better effect on liver repair, followed by the high dose. The islet cells of mice with glycosuria were repaired in varying degrees after treatment with the mulberry extract, and the islet cells were regenerated substantially. The high dose was the most effective. Regarding the effect on the kidney, the common section and PAS glycogen staining revealed that the high dose induced renal cell normalization. However, the TUNEL staining revealed that metformin better controlled the kidney cells, followed by the medium dose of the mulberry extract.

Thus, it was speculated that the main reason for hyperglycemia damage in the kidney is the glomerulus damaged by early inflammation. Metformin improved the blood glucose better than the extract, with faster control, and the renal cells recovered quickly.

The pathogenesis of chronic complications of DM is complicated, and apoptosis has been the focus in recent years [39]. In vitro studies have revealed that long-term hyperglycemia can increase β cell apoptosis and reduce the number of functional β cells. Thus, β cells stimulated by high blood glucose cannot produce sufficient insulin to maintain blood sugar levels in the normal range. Therefore, long-term hyperglycemia affects the expression of genes involved in apoptosis. Cell apoptosis is caused by diabetes in three ways [40]. One is the activation pathway of endogenous apoptosis mediated by mitochondria, another is the pathway of exogenous apoptosis activation mediated by death ligands and death receptors, and the third is the pathway of apoptosis activation mediated by the endoplasmic reticulum. Apoptosis is essential in the chronic complications of DM. However, the mechanism is not completely clarified. A certain degree of apoptosis is beneficial to repairing tissue function for normal insulin secretion.

## 5. Conclusions

In summary, mulberry leaf extract significantly attenuated streptozotocin (STZ)-induced diabetes. Hence, mulberry leaf can be used to develop new hypoglycemic drugs or functional health foods with wide applicability. This experiment provides the pharmacological and pathological basis for clinically preventing and treating diabetes with mulberry leaf water extract. Moreover, the effective hypoglycemic ingredient in the mulberry leaf water extract is also worth further research and development.

## Figures and Tables

**Table 1:** The effects of aqueous mulberry leaf extract on glucose tolerance in T1DM mice (x ± s).

Group	Dose (mg·kg^−1^)	N	Blood Glucose/(mmol·L^−1^)				AUC (mmol·h/L)
			0 h	0.5 h	1 h	2 h	
Control group		20	5.4 ± 0.4 **	14.7 ± 4.3 **	9.7 ± 1.9 **	5.6 ± 1.0 **	18.8 ± 4.0 **
Model group		20	24.9 ± 2.8	31.6 ± 1.2	29.6 ± 1.4	27.6 ± 1.6	57.9 ± 2.4
Metformin group	270	20	25.4 ± 3.0	27.4 ± 2.6 **	21.7 ± 2.5 **	19.1 ± 2.2 **	45.9 ± 4.7 **
Treatment groups (High-dose, Medium-dose, Low-dose)	1200	20	25.3 ± 2.5	30.0 ± 1.5	26.0 ± 3.0 **	23.0 ± 2.5 **	52.4 ± 4.5 **
	600	20	24.3 ± 3.8	30.0 ± 1.6	26.8 ± 2.1 *	24.7 ± 2.1 *	53.6 ± 4.1 *
	300	20	24.9 ± 2.0	30.4 ± 2.0	27.5 ± 1.7	25.1 ± 1.8 *	54.7 ± 3.4

* p < 0.05, ** *p* < 0.01 vs. model group.

**Table 2:** Effects of aqueous mulberry leaf extract on SOD activity and MDA level in serum of T1DM mice (x ± s).

Group	Dose (mg·kg^−1^)	N	SOD (U·mL^−1^)	MDA (nmol·mL^−1^)
Control group		20	154.59 ± 36.66 **	4.59 ± 2.67 **
Model group		20	113.78 ± 22.20	8.14 ± 1.62
Metformin group	270	20	144.53 ± 21.90 **	5.00 ± 2.73 **
Treatment groups (High-dose, Medium-dose, Low-dose)	1200	20	148.87 ± 18.36 **	4.42 ± 1.96 **
	600	20	142.99 ± 20.41 **	4.99 ± 2.12 **
	300	20	139.19 ± 16.11 *	5.29 ± 2.60 *

* p < 0.05; ** *p* < 0.01 vs. model group.

## Data Availability

Data is contained within the article.

## Supplementary Materials

The following supporting information can be downloaded at: https://www.mdpi.com/article/10.3390/cimb45070343/s1, Table S1: Statistical analysis of the effects of aqueous mulberry leaf extract on ALP in T1DM mice; Table S2: Statistical analysis of the effects of aqueous mulberry leaf extract on ALT in T1DM mice; Table S3: Statistical analysis of the effects of aqueous mulberry leaf extract on AST in T1DM mice; Table S4: Statistical analysis of the effects of aqueous mulberry leaf extract on BUN in T1DM mice; Table S5 Statistical analysis of the effects of aqueous mulberry leaf extract on CHO in T1DM mice; Table S6 Statistical analysis of the effects of aqueous mulberry leaf extract on LDH in T1DM mice; Table S7 Statistical analysis of the effects of aqueous mulberry leaf extract on β-2-GM in T1DM mice. Table S8: The biochemical parameters observed in all groups of mice.

## Abbreviations

Diabetes mellitus (DM), alkaline phosphatase (ALP), alanine aminotransferase (ALT), aspartate transaminase (AST), blood urea nitrogen (BUN), gamma-glutamyl transferase (GGT), glucose (GLU), high-density lipoprotein cholesterol (HDL-C), lactate dehydrogenase (LDH), low-density lipoprotein cholesterol (LDL-C), periodic acid Schiff (PAS), streptozotocin (STZ), malonic dialdehyde (MDA), type 1 diabetes mellitus (T1DM), cholesterol (CHO), triglyceride (TG), total serum cholesterol (TC), least significant difference (LSD).
