# Supplementary material for: Pharmacological and Pathological Effects of Mulberry Leaf Extract on the Treatment of Type 1 Diabetes Mellitus Mice
Source: Curr Issues Mol Biol. 2023 Jun 29;45(7):5403–21. doi: 10.3390/cimb45070343 (PMC10378407; doi:10.3390/cimb45070343)
Supplement: Supplementary file 1 [file cimb-45-00343-s001.zip › Supplementary Information (revise).pdf]

Supplementary Material

Pharmacological and Pathological Effect of Mulberry Leaf Extract on the Treatment of Type 1 Diabetes Mellitus Mice

Liru Luo [1,2], Wei Fan[1,2], Jingping Qin[2,3], Shiyin Guo[1,2], Hang Xiao[1,2], and Zhonghai Tang [1,2,*]

*** Correspondence:** Zhonghai Tang: tangzh@hunau.edu.cn

Supplementary Tables

Table S1. Statistical analysis of the effects of mulberry leaf aqueous extract on ALP in T1DM mice

Group	Mean deviation (I-J)	Standard error	Statistical Significance	95% Confidence Interval of the Difference	
				Lower	Upper
Treatment groups(High-dose , Middle-dose, Low-dose)	82.8000***	16.94337	.000	48.8306	116.7694
	42.3000*	16.94337	.016	8.3306	76.2694
	50.4000**	16.94337	.004	16.4306	84.3694
Metformin group	32.0000	16.94337	.064	-1.9694	65.9694
Control group	119.3260***	16.94337	.000	85.3566	153.2954

*$P < 0.05$, **$P < 0.01$,*** P＜0.001*vs* model group

Table S2. Statistical analysis of the effects of mulberry leaf aqueous extract on ALT in T1DM mice

Group	Mean	Standard	Statistical	95% Confidence Interval

	deviation (I-J)	error	Significance	of the Difference	
				Lower	Upper
Treatment groups(High-dose, Middle-dose, Low-dose)	56.10000*	23.41061	.021	8.9486	103.2514
	62.05556*	23.89335	.013	13.9319	110.1792
	58.00000*	24.48340	.022	8.6879	107.3121
Metformin group	20.40000	23.41061	.388	-26.7514	67.5514
Control group	80.25000**	24.48340	.002	30.9379	129.5621

* P＜0.05， ** P＜0.01*vs* model group(

Table S3. Statistical analysis of the effects of mulberry leaf aqueous extract on AST in T1DM mice

Group	Mean deviation (I-J)	Standard error	Statistical Significance	95% Confidence Interval of the Difference	
				Lower	Upper
Treatment groups(High-dose, Middle-dose, Low-dose)	106.03333**	31.14303	.001	43.3080	168.7586
	89.33333**	31.78522	.007	25.3146	153.3521
	113.70833**	32.57016	.001	48.1087	179.3080
Metformin group	69.83333*	31.14303	.030	7.1080	132.5586
Control group	136.20833***	32.57016	.000	70.6087	201.8080

*P＜0.05， ** P＜0.01， *** P＜0.001*vs* model group

Table S4. Statistical analysis of the effects of mulberry leaf aqueous extract on BUN in T1DM mice

Group	Mean deviation (I-J)	Standard error	Statistical Significance	95% Confidence Interval of the Difference	
				Lower	Upper
Treatment groups(High-dose, Middle-dose, Low-dose)	9.30767**	2.89245	.002	3.4820	15.1334
	9.82944**	2.95209	.002	3.8836	15.7753
	12.98792***	3.02499	.000	6.8953	19.0806
Metformin group	11.86167***	2.89245	.000	6.0360	17.6874
Control group	16.82042***	3.02499	.000	10.7278	22.9131

*P＜0.05，** P＜0.01， *** P＜0.001*vs* model group

Table S5. Statistical analysis of the effects of mulberry leaf aqueous extract on CHO in T1DM mice

Group	Mean deviation (I-J)	Standard error	Statistical Significance	95% Confidence Interval of the Difference	
				Lower	Upper
Treatment groups(High-dose , Middle-dose, Low-dose)	7.47867***	1.37165	.000	4.7160	10.2413
	7.50333***	1.39993	.000	4.6837	10.3229
	7.96917***	1.43451	.000	5.0799	10.8584
Metformin group	7.76367***	1.37165	.000	5.0010	10.5263
Control group	8.13667***	1.43451	.000	5.2474	11.0259

*P＜0.05， ** P＜0.01， *** P＜0.001*vs* model group

Table S6. Statistical analysis of the effects of mulberry leaf aqueous extract on LDH in T1DM mice

Group	Mean deviation (I-J)	Standard error	Statistical Significance	95% Confidence Interval of the Difference	
				Lower	Upper
Treatment groups(High-dose, Middle-dose, Low-dose)	854.96667***	204.45670	.000	443.1697	1266.7636
	685.61111**	208.67274	.002	265.3226	1105.8996
	987.16667***	213.82593	.000	556.4991	1417.8342
Metformin group	783.56667***	204.45670	.000	371.7697	1195.3636
Control group	683.54167**	213.82593	.003	252.8741	1114.2092

*P＜0.05， ** P＜0.01， *** P＜0.001*vs* model group

Table S7. Statistical analysis of the effects of mulberry leaf aqueous extract on β-2-GM in T1DM mice

Group	Mean deviation (I-J)	Standard error	Statistical Significance	95% Confidence Interval of the Difference	
				Lower	Upper
Treatment groups(High-dose, Middle-dose, Low-dose)	40.6103433***	6.1976219	.000	27.915090	53.305596
	43.2125933***	6.1976219	.000	30.517340	55.907846
	42.8663600***	6.1976219	.000	30.171107	55.561613

Metformin group	41.4746100***	6.1976219	.000	28.779357	54.169863
Control group	36.5227800***	6.4732050	.000	23.263021	49.782539

*P＜0.05，** P＜0.01， *** P＜0.001*vs* model group

Table S8. The biochemical parameters observed in all groups of mice (x±s)

Group		Treatment groups			Metformin group	Model group	Control group
		High-dose	Middle-dose	Low-dose			
Fasting blood glucose	0d	24.20±3.80	25.90±3.40	24.00±5.70	25.10±3.40	24.70±5.50	6.10±0.90
	10d	21.50±2.40	23.40±5.90	21.70±3.00	20.30±1.80	25.60±3.50	6.10±0. 90
	20d	19.50±4.30	21.30±5.80	20.90±4.90	17.60±1.60	25.10±7.00	5.50±0. 90
	30d	17.10±3.60	21.20±3.50	20.70±3.40	14.30±2.10	25.30±4.40	5.80±0. 80
	40d	15.20±2.20	18.10±3.00	18.60±3.30	12.60±1.80	26.20±3.20	5.80±1.10
Blood glucose(AUC)		52.40±4.50	53.60±4.10	54.70±3.40	45.90±4.70	57.90±2.40	18.80±4.00
HDL-C		3.02±0.67	2.75±0.55	2.75±0.32	3.32±0.78	2.14±0.36	3.31±0.54
LDL-C		0.41±0.06	0.42±0.05	0.44±0. 10	0.41±0.05	0.50±0.13	0.21±0.03
TG		1.85±0.50	2.05±0.53	2.09±0.36	1.74±0.38	2.41±0.64	1.06±0.32
Ins		2.94±0.24	2.89 ±0.31	2.65±0.27	2.00±0.31	1.67±0.69	3.96 ±0.50
ALP		101.40±6.57	141.90±12.51	133.80±12.03	152.20±13.08	184.20±16.82	64.87±7.92
ALT		72.40±6.79	66.44±9.20	70.50±6.29	108.10±17.28	128.50±41.80	48.25±3.59
AST		123.30±13.41	140.00±11.53	115.63±8.93	159.50±26.09	229.33±47.26	93.13±9.59
BUN		14.65±1.81	14.13±1.80	10.97±1.24	12.10±1.64	23.96±4.27	7.14±0.33
CHO		3.46±0.19	3.43±0.26	2.97±0.13	3.17±0.29	10.94±3.16	2.80±0.11
LDH		1190.20±93.96	1359.56±93.02	1058.00±70.63	1261.60±63.11	2045.17±280.27	1361.63±217.22
β-2-GM		25.03±2.35	22.43±1.05	22.78±2.46	24.17±1.17	65.65±10.61	29.12±3.61
