# Supplementary material for: Pharmacological and Pathological Effects of Mulberry Leaf Extract on the Treatment of Type 1 Diabetes Mellitus Mice
Source: Curr Issues Mol Biol. 2023 Jun 29;45(7):5403–21. doi: 10.3390/cimb45070343 (PMC10378407; doi:10.3390/cimb45070343)
Supplement: Supplementary file 1 [file cimb-45-00343-s001.zip › Supplementary materials (raw data).pdf]

Supplementary Material(Raw Data)

Pharmacological and Pathological Effect of Mulberry Leaf Extract on the Treatment of Type 1 Diabetes Mellitus Mice

Liru Luo [1,2], Wei Fan[1,2], Jingping Qin[2,3], Shiyin Guo[1,2], Hang Xiao[1,2], and Zhonghai Tang [1,2,*]

*** Correspondence:** Zhonghai Tang: tangzh@hunau.edu.cn

Raw Data

Figure 1 raw data : Hypoglycemic effect of the aqueous mulberry leaf extract on T1DM mice (x±s)

**Group**	**Dose** (mg·kg^{-1})	**N**	**Fasting blood glucose** (mmol·L^{-1})				
			0d	10d	20d	30d	40d
Control group		20	6.1±0.9**	6.1±0. 9**	5.5±0. 9**	5.8±0. 8**	5.8±1.1**
Model group		20	24.7±5.5	25.6±3.5	25.1±7.0	25.3±4.4	26.2±3.2
Metformin group	270	20	25.1±3.4	20.3±1.8**	17.6±1.6**	14.3±2.1**	12.6±1.8**
Treatment groups(High-dose, Middle-dose, Low-dose)	1200	20	24.2±3.8	21.5±2.4*	19.5±4.3*	17.1±3.6**	15.2±2.2**
	600	20	25.9±3.4	23.4±5.9	21.3±5.8	21.2±3.5*	18.1±3.0**
	300	20	24.0±5.7	21.7±3.0*	20.9±4.9	20.7±3.4**	18.6±3.3**

**P* < 0.05 , ***P* < 0.01 *vs* model group

Figure 2 raw data: Effect of the aqueous mulberry aqueous leaf extract on serum lipids (HDL-C, LDL-C, and TG) and serum insulin levels in T1DM mice.(x±s)

**Group**	**Dose**(mg·kg^{-1})	**N**=20	HDL-C (mmol·L^{-1})	LDL-C (mmol·L^{-1})	TG (mmol·L^{-1})	Ins (mIU·L^{-1})
Control group		20	3.31±0.54**	0.21±0.03**	1.06±0.32**	3.96 ±0.50**
Model group		20	2.14±0.36	0.50±0.13	2.41±0.64	1.67±0.69
Metformin group	270	20	3.32±0.78**	0.41±0.05*	1.74±0.38**	2.00±0.31

Treatment groups(High-dose, Middle-dose, Low-dose)	1200<br>600<br>300	20<br>20<br>20	3.02±0.67**<br>2.75±0.55*<br>2.75±0.32*	0.41±0.06*<br>0.42±0.05<br>0.44±0. 10	1.85±0.50*<br>2.05±0.53<br>2.09±0.36	2.94±0.24**<br>2.89 ±0.31**<br>2.65±0.27**

*P < 0.05 ; **P < 0.01 *vs* model group

Figure 3 raw data: The effects of the aqueous mulberry leaf extract on ALP in T1DM mice (x±s)

Group	N	Mean	Mean deviation	Standard error	95% Confidence Interval of the Difference		minimum	maximum
					Lower	Upper		
Treatment groups(High -dose, Middle-dose, Low-dose)	20	101.4000	20.76964	6.56794	86.5423	116.2577	65.00	134.00
	20	141.9000	39.55432	12.50818	113.6045	170.1955	85.00	201.00
	20	133.8000	38.04325	12.03033	106.5855	161.0145	67.00	179.00
Metformin group	20	152.2000	41.36504	13.08077	122.6092	181.7908	84.00	198.00
Model group	20	184.2000	53.20150	16.82379	146.1419	222.2581	150.00	314.00
Control group	20	64.8740	25.05300	7.92245	46.9522	82.7958	12.00	103.00
Total	120	129.7290	52.71693	6.80573	116.1108	143.3472	12.00	314.00

Figure 4 raw data: The effects of the aqueous mulberry leaf extract on ALT in T1DM mice

Group	N	Mean	Mean deviation	Standard error	95% Confidence Interval of the Difference		minimum	maximum
					Lower	Upper		
Treatment groups(High-dose, Middle-dose, Low-dose)	20	72.4000	21.47971	6.79248	57.0343	87.7657	52.00	118.00
	20	66.4444	27.61391	9.20464	45.2185	87.6704	6.00	95.00
	20	70.5000	17.80048	6.29342	55.6184	85.3816	50.00	99.00
Metformin group	20	108.1000	54.65742	17.28419	69.0004	147.1996	45.00	182.00
Model group	20	128.5000	102.37724	41.79533	21.0617	235.9383	58.00	335.00
Control group	20	48.2500	10.15241	3.58942	39.7624	56.7376	35.00	66.00
Total	120	80.8627	50.09472	7.01466	66.7734	94.9521	6.00	335.00

Figure 5 raw data: The effects of the aqueous mulberry leaf extract on AST in T1DM mice

Group	N	Mean	Mean deviation	Standard error	95% Confidence Interval of the Difference		minimum	maximum
					Lower	Upper		
Treatment groups(High-dose, Middle-dose, Low-dose)	20	123.3000	42.40820	13.41065	92.9630	153.6370	83.00	202.00
	20	140.0000	34.59407	11.53136	113.4086	166.5914	93.00	206.00
	20	115.6250	25.26962	8.93416	94.4991	136.7509	80.00	158.00
Metformin group	20	159.5000	82.51902	26.09481	100.4694	218.5306	79.00	307.00
Model group	20	229.3333	115.76298	47.26004	107.8475	350.8191	103.00	443.00
Control group	20	93.1250	27.12109	9.58875	70.4512	115.7988	62.00	133.00
Total	120	139.8824	69.35031	9.71099	120.3773	159.3874	62.00	443.00

Figure 6 raw data: The effects of the aqueous mulberry leaf extract on BUN in T1DM mice

Group	N	Mean	Mean deviation	Standard error	95% Confidence Interval of the Difference		minimum	maximum
					Lower	Upper		
Treatment groups(High-dose, Middle-dose,	20	14.6540	5.73807	1.81454	10.5492	18.7588	9.47	27.24
	20	14.1322	5.41062	1.80354	9.9733	18.2912	8.90	27.54

Low-dose)	20	10.9737	3.50236	1.23827	8.0457	13.9018	8.80	19.48
Metformin group	20	12.1000	5.19927	1.64415	8.3807	15.8193	8.40	24.88
Model group	20	23.9617	10.45127	4.26671	12.9937	34.9296	11.05	36.56
Control group	20	7.1413	.92370	.32658	6.3690	7.9135	6.10	8.86
Total	120	13.4004	7.04133	.98598	11.4200	15.3808	6.10	36.56

Figure 7 raw data: The effects of the aqueous mulberry leaf extract on CHO in T1DM mice

Group	N	Mean	Mean deviation	Standard error	95% Confidence Interval of the Difference		minimum	maximum
					Lower	Upper		
Treatment groups(High-dose, Middle-dose, Low-dose)	20	3.4580	.60835	.19238	3.0228	3.8932	2.56	4.22
	20	3.4333	.78626	.26209	2.8290	4.0377	2.40	4.67
	20	2.9675	.36636	.12953	2.6612	3.2738	2.49	3.43
Metformin group	20	3.1730	.92535	.29262	2.5110	3.8350	2.06	4.93
Model group	20	10.9367	7.74456	3.16170	2.8093	19.0641	3.09	23.72
Control group	20	2.8000	.31108	.10998	2.5399	3.0601	2.38	3.36
Total	120	4.0975	3.57331	.50036	3.0924	5.1025	2.06	23.72

Figure 8 raw data: The effects of the aqueous mulberry leaf extract on LDH in T1DM mice

Group	N	Mean	Mean	Standard	95% Confidence Interval	minim	maximu

			deviation	error	of the Difference		um	m
					Lower	Upper		
Treatment groups(High-dose, Middle-dose, Low-dose)	20	1190.2000	297.13214	93.96143	977.6445	1402.7555	761.00	1666.00
	20	1359.5556	279.05246	93.01749	1145.0568	1574.0543	862.00	1761.00
	20	1058.0000	199.77988	70.63285	890.9798	1225.0202	698.00	1335.00
Metformin group	20	1261.6000	199.56185	63.10700	1118.8421	1404.3579	962.00	1528.00
Model group	20	2045.1667	686.52412	280.27230	1324.7038	2765.6295	1204.00	2907.00
Control group	20	1361.6250	614.38051	217.21631	847.9900	1875.2600	895.00	2800.00
Total	120	1340.8235	468.33632	65.58020	1209.1018	1472.5452	698.00	2907.00

Figure 9 raw data: The effects of the aqueous mulberry leaf extract on β-2-GM in T1DM mice

Group	N	Mean	Mean deviation	Standard error	95% Confidence Interval of the Difference		minimum	maximum
					Lower	Upper		
Treatment groups(High-dose, Middle-dose, Low-dose)	20	25.034117	5.7495592	2.3472477	19.000324	31.067909	19.9338	34.5004
	20	22.431867	2.5773947	1.0522170	19.727057	25.136677	19.1547	26.7910
	20	22.778100	6.0248171	2.4596213	16.455442	29.100758	18.6272	34.7519
Metformin group	20	24.169850	2.8596068	1.1674296	21.168877	27.170823	20.1367	28.1787

Model group	20	65.644460	23.7278046	10.6113968	36.182499	95.106421	25.5494	87.1828
Control group	20	29.121680	8.0661501	3.6072920	19.106232	39.137128	18.2540	35.8393
Total	120	30.597479	17.6549616	3.0278009	24.437372	36.757587	18.2540	87.1828
